# Ganglioside Alters Phospholipase Trafficking, Inhibits NF-κB Assembly, and Protects Tight Junction Integrity

**DOI:** 10.3389/fnut.2021.705172

**Published:** 2021-07-05

**Authors:** John J. Miklavcic, Qun Li, Jordan Skolnick, Alan B. R. Thomson, Vera C. Mazurak, Micheal Tom Clandinin

**Affiliations:** ^1^Schmid College of Science and Technology, Chapman University, Orange, CA, United States; ^2^School of Pharmacy, Chapman University, Irvine, CA, United States; ^3^Agricultural, Food & Nutritional Science, University of Alberta, Edmonton, AB, Canada; ^4^Division of Gastroenterology, Western University, London, ON, Canada; ^5^Faculty of Medicine & Dentistry, University of Alberta, Edmonton, AB, Canada

**Keywords:** crohn, nutrition, ulcerative colitis, sphingolipid, immunology, inflammatory bowel disease, inflammation, intestinal permeability

## Abstract

**Background and Aims:** Dietary gangliosides are present in human milk and consumed in low amounts from organ meats. Clinical and animal studies indicate that dietary gangliosides attenuate signaling processes that are a hallmark of inflammatory bowel disease (IBD). Gangliosides decrease pro-inflammatory markers, improve intestinal permeability, and reduce symptoms characteristic in patients with IBD. The objective of this study was to examine mechanisms by which dietary gangliosides exert beneficial effects on intestinal health.

**Methods:** Studies were conducted *in vitro* using CaCo-2 intestinal epithelial cells. Gangliosides were extracted from milk powder and incubated with differentiated CaCo-2 cells after exposure to pro-inflammatory stimuli. Gut barrier integrity was assessed by electron microscopy, epithelial barrier function was examined by measuring transepithelial electric resistance, and content of HBD-2, IL-23, NF-κB, and sPLA_2_ was assessed by ELISA.

**Results:** Ganglioside attenuated the decrease in integrity of tight junctions induced by pro-inflammatory stimuli and improved epithelial barrier function (*P* < 0.05). Ganglioside decreased the basolateral secretion of sPLA_2_ (*P* ≤ 0.05), lowered HBD-2 and IL-23 levels (*P* ≤ 0.05), and inhibited NF-κB activation (*P* ≤ 0.05).

**Conclusions:** In summary, the present study indicates that ganglioside GD3 improves intestinal integrity by altering sPLA_2_ trafficking, and the production of pro-inflammatory mediators is mitigated by decreasing assembly of the NF-κB complex. Dietary gangliosides may have promising potential beneficial effects in IBD as decreased inflammatory signaling, improved intestinal integrity, and maintenance of epithelial barrier function have been demonstrated *in vitro*.

## Introduction

Inflammatory bowel disease (IBD) is a chronic, relapsing disorder resulting from persistent inflammation affecting the mucosa of the gastrointestinal tract (1). Management of Crohn disease (CD) and ulcerative colitis (UC) is challenging and may consist of drug treatments including glucocorticosteroids, immunosuppressants, and/or biologics. Some individuals with IBD do not respond to drug treatment, while others experience adverse effects associated with treatment (2). Failure to maintain remission is common, thus new treatment initiatives are urgently needed.

Individuals consuming diet abundant in anti-inflammatory bioactives have a reduced risk for CD (3). Gangliosides are a class of polar lipids abundantly expressed in the nervous system and found ubiquitously in tissues and body fluids, including human milk (4). Ingestion of dietary ganglioside was shown to be safe at 43 mg/day for 8 weeks in adults (5). The bioavailability of ganglioside has been demonstrated as plasma concentration of ganglioside GD3 increased 40% within 2 weeks of supplementation with ganglioside in healthy adults and in adults with IBD (5). Dietary gangliosides consumed from a complex milk-lipid fraction reduced intestinal permeability by 19% in healthy adults, and also improved emotional health and systemic symptoms in a pilot study of five participants with IBD without a placebo control after 8 weeks of consumption (5). Dietary ganglioside is safe for consumption and has demonstrated clinical benefit in patients with IBD.

Reduction of ganglioside content is a hallmark of intestinal inflammation (2). The content of ganglioside catabolic enzymes beta-hexosaminidase and sialidase are elevated in the intestinal mucosa of patients with IBD relative to a control group (6), suggesting a depletion of ganglioside GD3 species in CD and UC. Exposure of infant bowel to ganglioside *ex vivo* reduced bowel necrosis and endothelin-1 production in response to lipopolysaccharide (LPS) (7). Dietary gangliosides increase cellular ganglioside content and decrease cholesterol content of microdomains in a rodent model (8). Reduced content of cholesterol in enterocyte membranes inhibits cellular entry of pathogens (9) and decreases the generation of pro-inflammatory signals (8). The mechanisms underlying the mitigation of pro-inflammatory signaling by dietary gangliosides remains largely unknown.

Intestinal epithelial cells are a key component to the physiologic barrier between the myriad of toxins, microbes, and antigens present in the gut lumen and the underlying mucosal immune system (10). Defects in the intestinal barrier and impaired immune function are characteristic of IBD. Disruption of intestinal barrier function results in increased permeability of the host gut to luminal antigens and bacteria, leading to the immune response and inflammation (11). Para-cellular permeability is regulated by a complex of macromolecules commonly described as adherens or tight junctions. IL-1β induces the expression of miR-200c-3p which causes a downregulation of the tight junction protein occludin (12). Dietary ganglioside has been reported to inhibit degradation of occludin in a preclinical model of IBD (13). Secretory phospholipase A_2_ (sPLA_2_) is a ubiquitous group of enzymes implicated in turnover of membrane phospholipids and lipid digestion. Activated sPLA_2_ catalyzes release of fatty acids from the sn-2 position of phospholipid in membranes, which is the first step in production of eicosanoids and other inflammatory mediators (14) that result in degradation of tight junction proteins.

Human beta-defensin (HBD)-2 coordinates innate and acquired immune responses (15) through nuclear factor-κB (NF-κB) (16). NF-κB is a primary regulator of inflammatory responses and plays a critical role in a variety of physiological and pathologic processes. Activation of NF-κB stimulates production of interleukin-23 (IL-23) (17). After binding to the IL-23 receptor, the production tumor necrosis factor alpha (TNF-α) is stimulated from T-cells thus contributing to the pathogenesis of IBD (18). It is crucial that efficacious therapies reduce TNF-α to induce clinical, endoscopic, and histopathological healing (19).

The focus of this study was to determine the potential mechanism by which ganglioside protects barrier integrity and function in a model of intestinal cell inflammation. It is hypothesized that ganglioside GD3 sustains barrier activity by decreasing secretion of sPLA_2_, inhibiting NF-κB activation, and mediating pro-inflammatory signaling.

## Methods

All authors had access to the study data and had reviewed and approved the final manuscript.

### Cell Culture

The human colon adenocarcinoma epithelial cell line CaCo-2 (American Type Culture Collection® HTB-37^TM^) was grown in complete medium (EMEM, Invitrogen), 10% (v/v) FBS, 1% (v/v) antibiotic/antimycotic, 26 mM sodium bicarbonate, 10 mM HEPES, 1 mM pyruvic acid, (37°C, 5% CO_2_). Cells were passaged at 90% confluence using 0.25% trypsin-2.65 mM EDTA and subcultured at a density of 1.6 × 10^4^ cells/cm^2^. All studies were performed on confluent cells of the CaCo-2 subclone between passages 20 and 40.

### Preparation of Ganglioside

Gangliosides were extracted from Zeta lipid-2 milk powder (Fonterra, Cambridge, New Zealand). Powder was added (1:60 w/v) to chloroform/methanol (C/M) (2:1 v/v), vortexed, and shaken (>2 h). Tubes were inversed several times after adding 0.025% (w/v) CaCl_2_/H_2_O. After centrifugation, the upper layer was withdrawn and applied to a Sep-Pak Classic C18 cartridge (Waters). Cartridges were rinsed with H_2_O before eluting ganglioside with methanol, followed by C/M (2:1 v/v). For quantification, aliquots (duplicate) were dried under N_2_ gas before H_2_O addition and vortex. Resorcinol-HCl was added to tubes before vortex and heating (160°C; 8 min). After cooling to room temperature, butylacetate/butanol (85:15 v/v) was added to tubes and vortexed. The upper layer was withdrawn and optical density was determined on a spectrophotometer (8452A, Hewlett Packard) at 580 nm. Total ganglioside was quantified as ganglioside-bound N-acetyl neuraminic acid with an N-acetyl neuraminic acid standard (SigmaAldrich). Gangliosides were dissolved in media (10 μg/ml) and all media were sonicated (130 W, 30 sec) prior to filter sterilization through a 0.2 μm syringe filter to facilitate dissolution of potential micelle formation.

### Stimulation of Cells

CaCo-2 cells were cultured at a density of 2.5 × 10^7^ cells/ml on 0.4 μm pore inserts in transwell plates (Corning Costar). The apical (upper) and basolateral (lower) compartments contained 0.5 and 2 ml of medium, respectively. After becoming confluent, cultures were then grown for 3 weeks to facilitate differentiation and expression of villi, microvilli, and small intestinal enterocyte function (20). Cells were divided into groups with the following experimental conditions for 48 h: grown in complete medium (control), incubated with ganglioside (10 μg/ml), exposure to LPS (100 μg/ml), or exposure to DSS (2.5%; Sigma Aldrich). Ganglioside was added to the culture medium 19 days after cultures became confluent. The cells were incubated for 24 h with LPS or for 48 h with DSS from the apical side 21 days after cultures became confluent. Culture media were collected and analyzed for HBD-2, IL-23, and sPLA_2_; and cells were harvested for analysis of NF-κB.

### Electron Microscopy

CaCo-2 cell monolayers were incubated in 2.5% (w/v) of glutaraldehyde/cacodylate buffer (pH 7.2) for 1.5 h and subsequently washed in this same buffer three times for 15 min before being fixed in 1% (v/v) osmium tetroxide buffer for 1.5 h at room temperature. Cells were then rinsed in distilled water; dehydrated in a series of 50, 70, and 90% absolute ethanol for 10 min. Samples were then embedded in Spurr's resin mixture (Spurr's resin/ethanol 50:50). Following polymerization at 70°C for 12 h, ultrathin sections were cut, stained with uranyl acetate and lead citrate, and viewed with a Hitachi H7000 Transmission Electron Microscope (Tokyo, Japan), or with a Hitachi Scanning Electron Microscope S2500 (Tokyo, Japan) using a method previously described (21) to examine the integrity of monolayers.

### TEER Assessment

Transepithelial electrical resistance (TEER) was assessed as an index of confluence, integrity and barrier function of cell monolayers (22). TEER of CaCo-2 monolayers grown on collagen-coated filter inserts was measured using a Volt Meter (World Precision Instruments, Hamden, CT). The TEER value measured in the absence of cells was used as background. The TEER of monolayers without added DSS or ganglioside represented the controls for each experiment. The TEER was measured in cell culture medium within one min of removing from incubator (37°C) before addition of pro-inflammatory stimulus (time zero) and then at time intervals and expressed as the ratio of the TEER at time t to the initial value (at time zero) for each series. Time course measurements were obtained independently from unique transwells.

### Measurement of Pro-inflammatory Markers and Immune Mediators

NF-κB activation was assessed using a TransAM NF-κB transcription factor family assay kit (Active Motif, Carlsbad, CA) according to the manufacturer's instructions. Human IL-23, HBD-2, and sPLA_2_ were assayed from cell supernatants using titerzyme ELISA Kits, according to the manufacturer's instructions (R&D Systems, Minneapolis, MN; Phoenix Pharmaceuticals Inc.; Cayman Chemicals, MI). A microplate reader (Eppendorf BioPhotometer) was used to determine optical density at 450 nm and corrected for background at 540 nm.

### Statistical Analysis

The study data may be made available to other researchers upon request. All experiments were independently replicated at least three times at different cell passages. Data are presented as mean ± SD. Statistical testing was conducted using an ANOVA or Duncan's multiple range test. There was no interaction between LPS and ganglioside when analyzed by a two-way ANOVA. An α value below 0.05 was considered statistically significant.

## Results

### Ganglioside Protected Intestinal Tight Junction and Adhesion Junction Proteins

**Figure 1:**
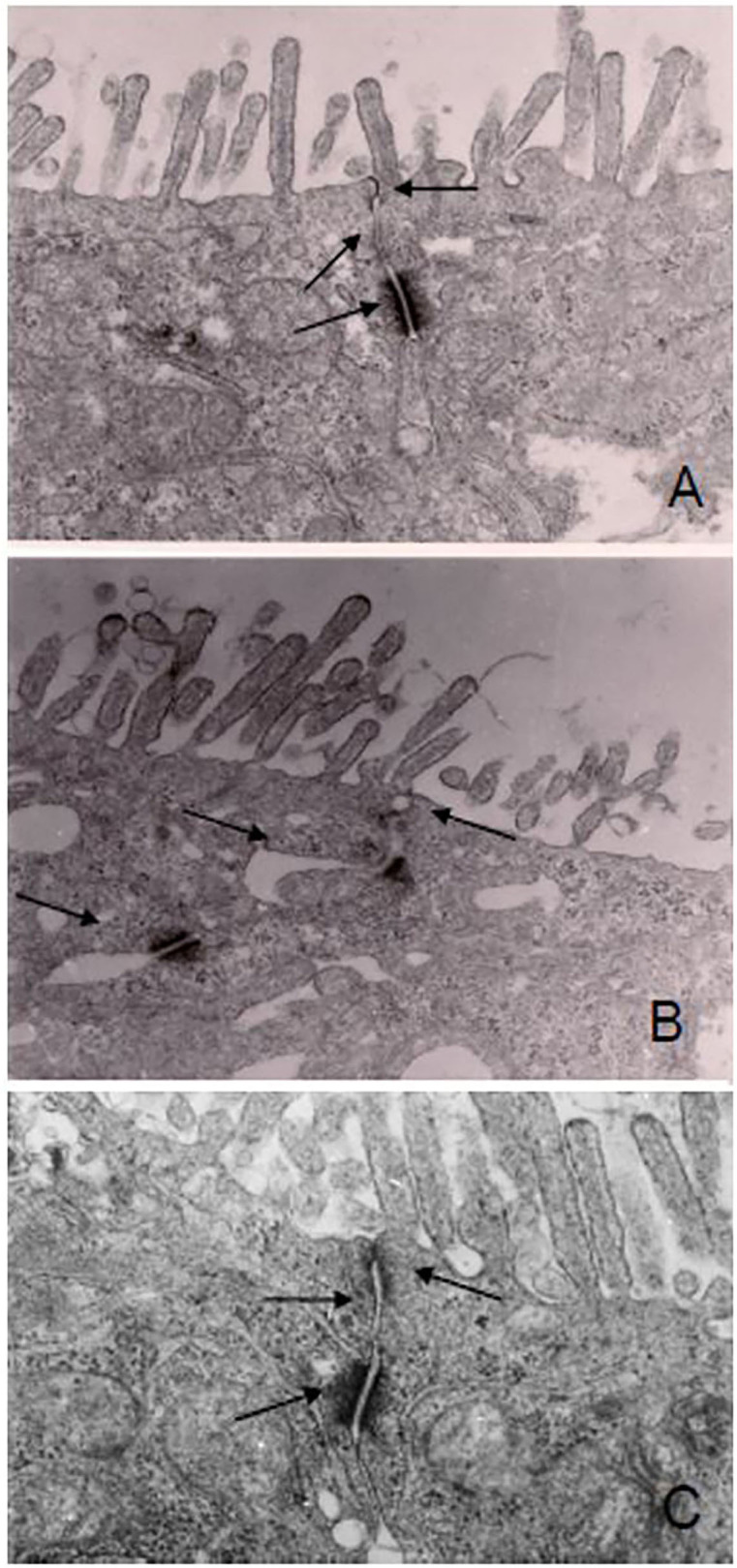
Ganglioside mitigates the disruption of junction complexes in CaCo-2 cells. Cell monolayers were exposed to pro-inflammatory stimulus with or without ganglioside pre-incubation. There was disruption of the tight junctions, adherens junction, and desmosomes in DSS-treated cells **(B)** compared with control cells **(A)** or cells pre-incubated with ganglioside **(C)**. Arrows indicate tight junction, adherens junction and desmosomes. Images were obtained at 2600x magnification on a Hitachi H7000 Transmission Electron Microscope.

**Figure 2:**
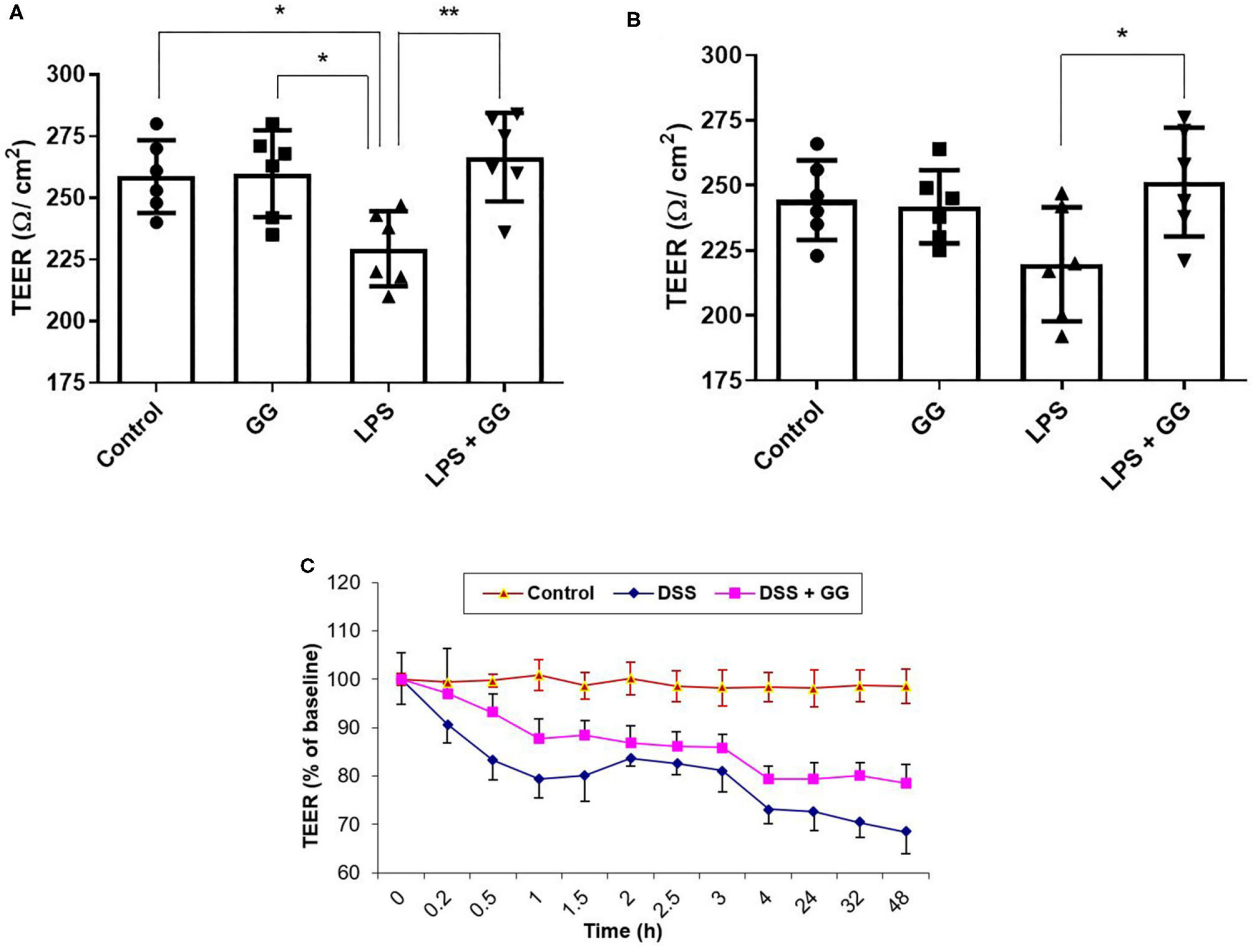
Ganglioside mitigates the reduction in TEER induced by pro-inflammatory stimuli. TEER was measured at **(A)** 8 h and **(B)** 24 h, and in a **(C)** 48 h timecourse after exposure to pro-inflammatory stimuli (*N* = 6 biological replicates in cell culture wells per group; *N* = 3 technical instrument reader replicates). For **(C)**, TEER measurement was 237 ± 5 Ω/cm^2^ at time = 0 h. Values illustrated the mean ± SD. Asterisks denotes statistical significance at *p < 0.05, **p < 0.01. In panel **(C)**, “DSS” significantly differs (*p* < 0.05) from “DSS + GG” at all-time points after 0 h. GG, ganglioside.

Visualization of CaCo-2 cell monolayer by electron microscopy revealed intact tight junctions, adherens junctions, and desmosomes in control cells (Figure 1A). DSS induced the opening and disruption of tight junctions and desmosomes (Figure 1B) which was attenuated by ganglioside (Figure 1C). Pre-incubation with ganglioside prevented the decrease in TEER induced by LPS and DSS (Figure 2).

### Ganglioside Regulates Phospholipase Activity

**Figure 3:**
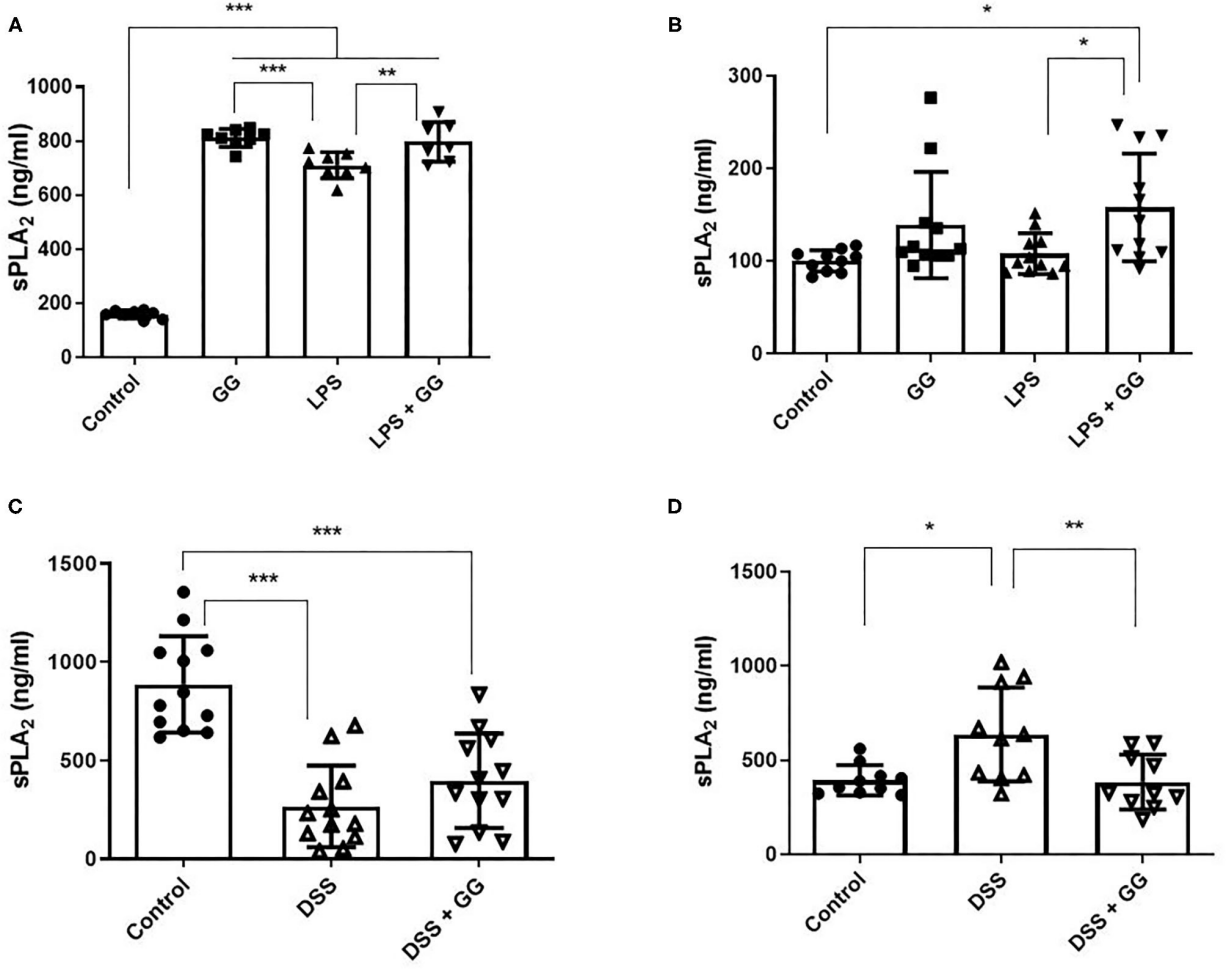
Secretory PLA_2_ activity was decreased by ganglioside at the basolateral membrane. CaCo-2 cells were grown on transwell inserts and incubated with ganglioside (10 μg/ml) for 48 h prior to exposure with inflammatory stimuli. **(A,C)** Illustrate the content of sPLA_2_ in apical medium and **(B,D)** illustrate the content of sPLA_2_ in basolateral medium (*N* = 8 biological replicates in cell culture wells per group; *N* = 3 technical instrument reader replicates). The mean ± SD is illustrated. Asterisks denote statistical significance at *p < 0.05, **p < 0.01, ***p < 0.001. GG, ganglioside.

The concentration of sPLA_2_ was elevated after incubation with LPS and ganglioside (Figures 3A,B). Pre-incubation with ganglioside mitigated the DSS-induced trafficking of sPLA_2_ to the basolateral membrane (Figures 3C,D).

### Ganglioside Decreased Pro-inflammatory Cytokine Levels

**Figure 4:**
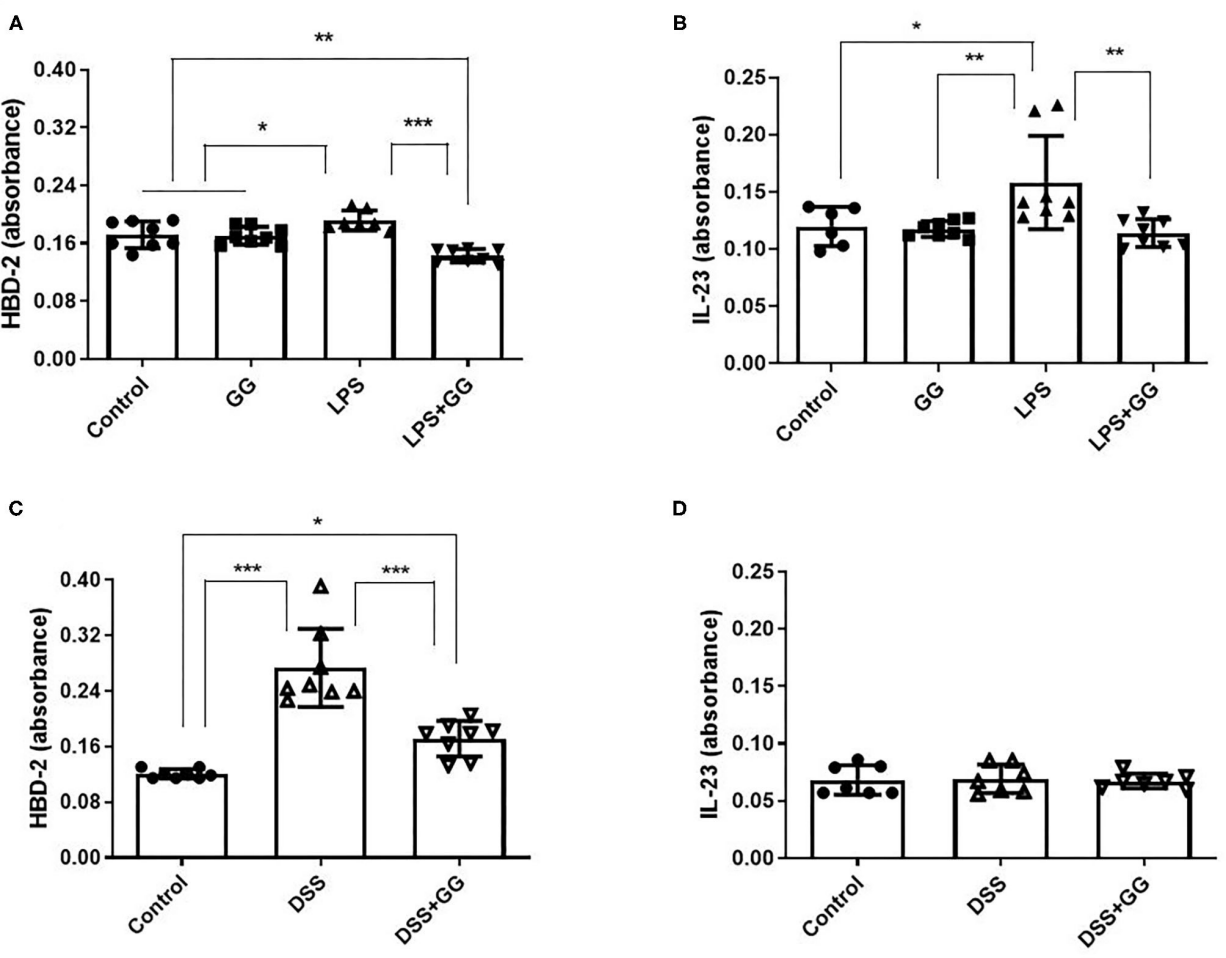
The content of HBD-2 and IL-23 is decreased by ganglioside. CaCo-2 cells grown on transwell inserts were incubated with ganglioside (10 μg/ml) for 48 h prior to exposure with LPS **(A,B)** or DSS **(C,D)**. Apical media were collected and analytes measured by ELISA (*N* = 8 biological replicates in cell culture wells per group; *N* = 3 technical instrument reader replicates). Bars represent the mean ± SD. Asterisks denote statistical significance at *p < 0.05, **p < 0.01, ***p < 0.005. GG, ganglioside.

**Figure 5:**
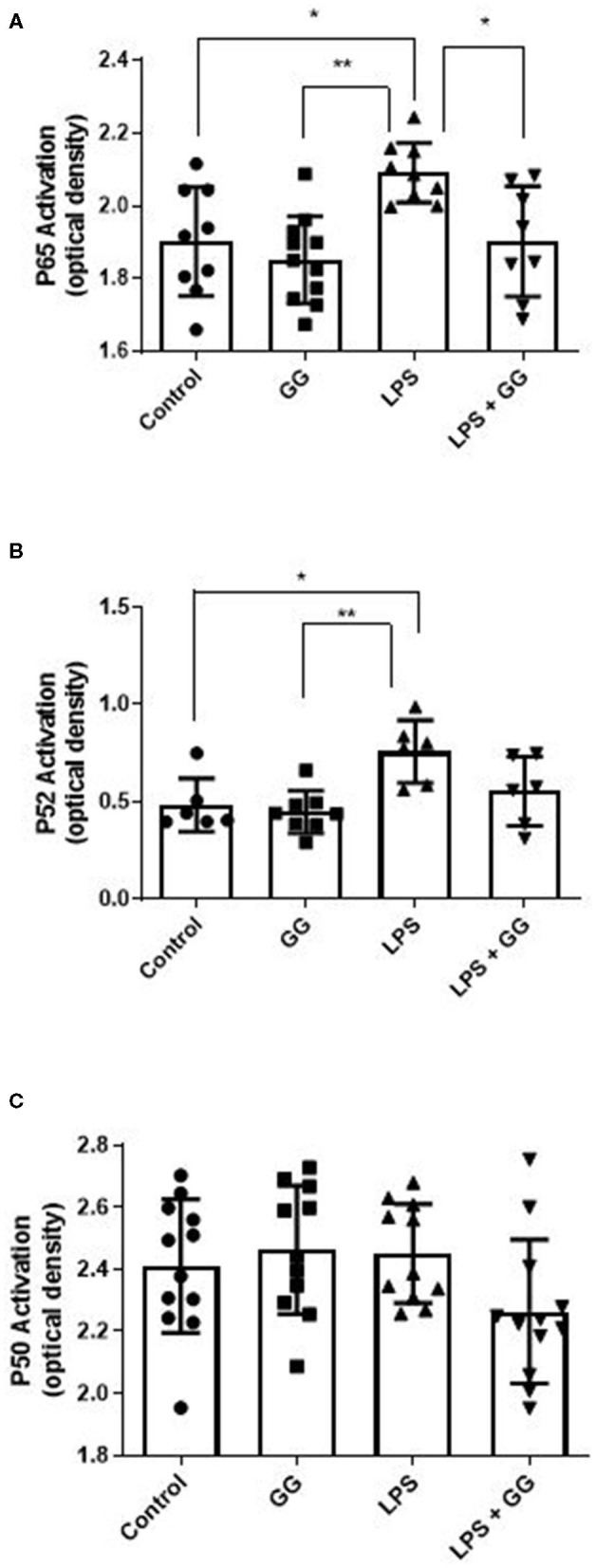
Ganglioside inhibits LPS-stimulated activation of NF-κB P65 and P52 subunits. CaCo-2 cells were incubated with ganglioside (10 μg/ml) for 48 h prior to exposure with inflammatory stimuli. Gangliosides mitigates the LPS-induced increase in subunits P65 **(A)** and P52 **(B)** but not P50 **(C)**, indicating that NF-κB complex formation and translocation is inhibited by ganglioside. Values illustrated are means ± SD. (*N* = 6 biological replicates in cell culture wells per group; *N* = 3 technical instrument reader replicates). The asterisk denotes statistical significance at *p < 0.05, **p < 0.01. GG, ganglioside.

LPS stimulation increased HBD-2 and IL-23 in the apical media from differentiated CaCo-2 cell monolayers (Figure 4). Incubation with ganglioside alone did not affect HBD-2 concentration compared to control. Pre-incubation with ganglioside mitigated the increases in HBD-2 and IL-23 content induced by pro-inflammatory stimulus (Figure 4). Incubation with pro-inflammatory stimuli significantly increased NF-κB activation in CaCo-2 cells (Figure 5). Pre-incubation with ganglioside reduced LPS-mediated activation of NF-κB P65 and P52 subunits (Figures 5A,B), but not P60 subunit (Figure 5C).

## Discussion

**Figure 6:**
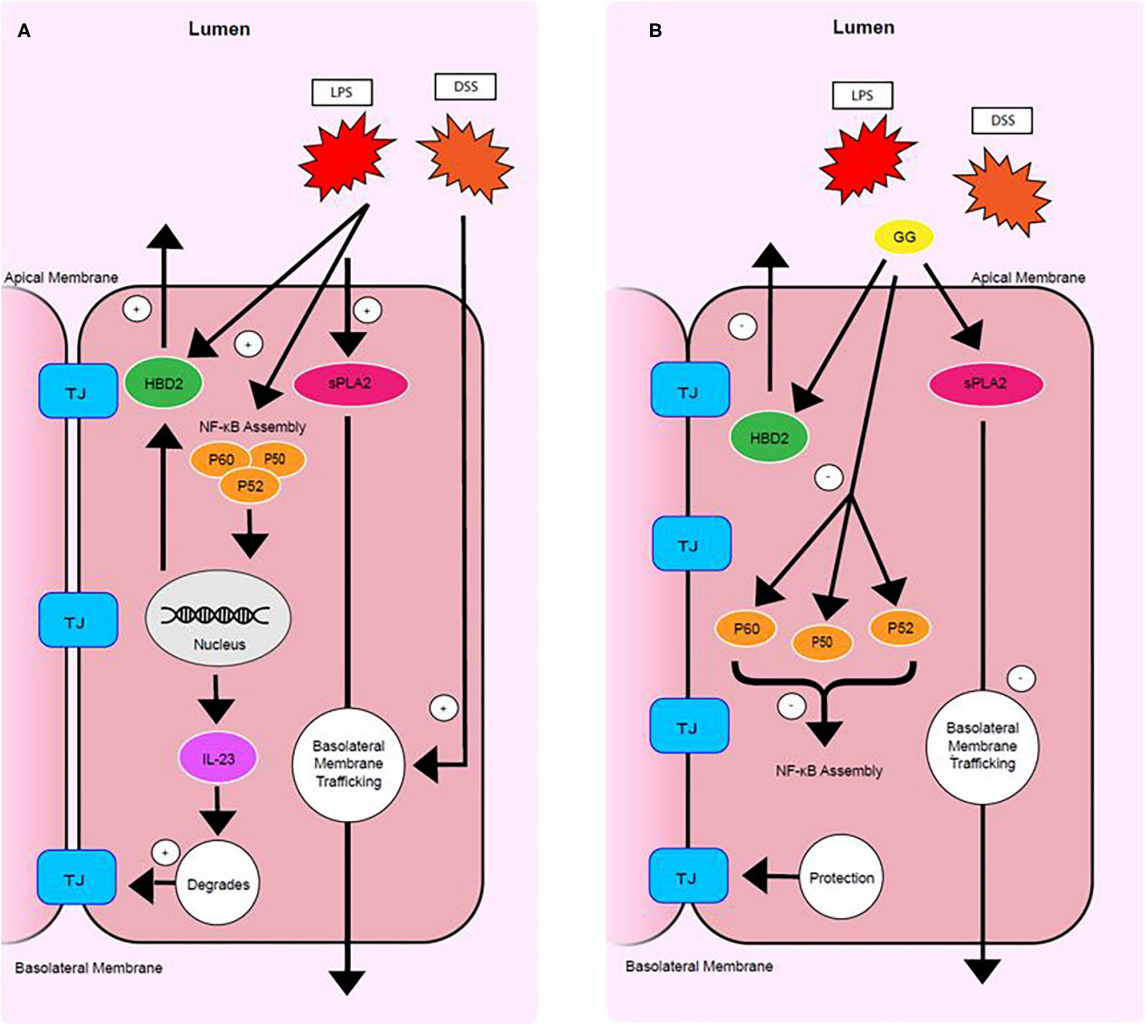
Mode of action of ganglioside. Pro-inflammatory stimuli upregulate sPLA_2_ trafficking, HBD-2 production, NF-κB subunit production and assembly, IL23 signaling, and tight junction degradation **(A)**. Ganglioside protects mucosal integrity by (1) inhibiting secretion of HBD-2, (2) decreasing NF-κB assembly, (3) lowering IL-23 production, and by (4) inhibiting trafficking of sPLA_2_ to the basolateral membrane **(B)**. TJ. tight junction.

The present study demonstrated that the disruption of intestinal tight junction and adhesion junction and increase in intestinal permeability induced by pro-inflammatory stimuli could be mitigated by milk fat gangliosides consisting primarily of GD3 (Figure 6). This observation is consistent with findings in preclinical research models where dietary ganglioside inhibited degradation of tight junction protein occludin during LPS-induced acute inflammation in rats (13). Furthermore, bovine milk derived ganglioside mixture used in these experiments is similar to the composition of gangliosides present in human colostrum (23), predominantly GD3 (80%) with smaller amounts (20%) of GM3, GM1, and GD1a.

Secretory PLA_2_ activity results in a reduction in barrier integrity in CaCo-2 cells (24). Clinical research has shown sPLA_2_ to be increased in serum and colonic mucosa of patients with CD and UC (21). The present study suggests that LPS upregulates the concentration of sPLA_2_ and that DSS promotes trafficking of sPLA_2_ to the basolateral membrane (Figure 6). However, ganglioside GD3 promoted trafficking of sPLA_2_ to the apical membrane (Figure 6). Secretory PLA_2_ enzymes catalyze hydrolysis of the sn-2 position of membrane glycerophospholipids, leading to production of free fatty acids, mainly polyunsaturated fatty acids, and lysophospholipids. This reaction is of particular importance and depends on the fatty acid present in the sn-2 position. A long chain omega-3 fatty acid liberated at this position may be converted to pro-resolving mediators, but arachidonic acid may be converted to prostacyclin and bioactive eicosanoids including prostaglandins and leukotrienes. Dietary ganglioside downregulates production of pro-inflammatory mediators PGE_2_ and LTB_4_ in rats (25). Further research is needed to clarify how ganglioside regulates trafficking and secretion of sPLA_2_ at the apical and basolateral membranes and how the production of pro-inflammatory or pro-resolving lipid-derived mediators are affected.

Although enterocytes are targets of pathologic inflammation, enterocytes are also actively involved in immune tolerance (26) and generating an immune response at the mucosal level (27). Elevated levels of pro-inflammatory cytokines are observed in patients with IBD (28). HBD-2 and HBD-3 represent a link between innate and adaptive immune responses and are induced in intestinal dysbiosis (29) and during inflammation of CD and UC (30, 31). In the present study, ganglioside GD3 prevented the increase in IL-23 and secretion of HBD-2 induced by pro-inflammatory stimuli (Figure 6). Probiotic strains of bacteria can induce HBD-2 expression to function as a critical mediator of T-regulatory cell induction (32) and immune tolerance (33, 34). Taken together these data suggest that compromised immune tolerance in IBD caused by NOD2 and ATG16L1 variants (35) may be modulated by ganglioside to resolve mucosal inflammation independent of the presence of risk alleles.

Genetic polymorphisms of the IL-23 receptor (IL-23R) are frequently observed in IBD patients, signifying the importance of IL-23 and IL-23R signaling in IBD pathogenesis (36). Assembly of NF-κB facilitates IL-23 production (37) and the coordinated recruitment of Th17 cells (38). NF-κB has been identified as one of the key regulators in this immunological setting and central to the function of monocytes that exacerbate pro-inflammatory signaling. Macrophages and epithelial cells isolated from inflamed gut specimens from IBD patients showed augmented levels of NF-κB p65 (39). NF-κB subunits are kept inactive in the cytoplasm by an endogenous inhibitor protein of the IkB family (40). Under stimulation by IL-1 or LPS, IkB is phosphorylated, selectively ubiquitinated and rapidly degraded allowing NF-κB to translocate into the nucleus and induces target genes that have NF-κB binding domains (40). In IBD patients, increased NF-κB expression in mucosal macrophages is accompanied by increased capacity of these cells to produce and secrete TNF-α, IL-1, and IL-6 (39). In the present study, exposure of CaCo-2 cells to ganglioside GD3 mitigated the LPS-induced activation of NF-κB by attenuating production of the P65 and P52 subunits (Figure 6).

The lack of use of an anti-inflammatory control may be considered a limitation in this study. Whether the anti-inflammatory mode of action of gangliosides is similar to that of anti-inflammatory pharmaceuticals is unknow. In addition, only a single dose of ganglioside was used in the *in vitro* experiments. This was based on effective concentrations previously published (9) in addition to choosing a dose below the critical micellar concentration of gangliosides (41).

In summary, this study demonstrates that exposure of CaCo-2 cells to inflammatory stimuli produced some of the inflammatory responses characteristic of IBD. Exposure to ganglioside GD3 mitigated production of pro-inflammatory cytokines HBD-2 and IL-23 and this effect may be mediated by lowering NF-κB activation (Figure 6). Ganglioside GD3 mitigates the disruption of tight junctions induced by pro-inflammatory stimuli. Ganglioside protects intracellular phospholipid integrity by mitigating sPLA_2_ production and inhibiting sPLA_2_ trafficking to the basolateral membrane of enterocytes. Ganglioside may have potential therapeutic application either by inducing or maintaining remission in IBD as this research study has demonstrated downregulation of pro-inflammatory signaling *in vitro*.

## Data Availability Statement

The original contributions presented in the study are included in the article/supplementary material, further inquiries can be directed to the corresponding authors.

## Author Contributions

JM (data curation: equal, resources: supporting, visualization: equal, writing—review and editing: equal). QL (conceptualization: supporting, formal analysis: lead, investigation: lead, methodology: lead, visualization: equal, writing—original draft: lead). JS (data curation: supporting, visualization: equal). AT (supervision: supporting). VM (supervision: supporting). MC (conceptualization: lead, funding acquisition: lead, resources: lead, supervision: lead, writing—review and editing: equal). All authors contributed to the article and approved the submitted version.

## Conflict of Interest

The authors declare that the research was conducted in the absence of any commercial or financial relationships that could be construed as a potential conflict of interest.
